# Functional near-infrared spectroscopy to assess sensorimotor cortical activity during hand squeezing and ankle dorsiflexion in individuals with and without bilateral and unilateral cerebral palsy

**DOI:** 10.1117/1.NPh.7.4.045001

**Published:** 2020-10-06

**Authors:** Theresa Sukal-Moulton, Ana C. de Campos, Katharine E. Alter, Diane L. Damiano

**Affiliations:** a Northwestern University Feinberg School of Medicine, Department of Physical Therapy and Human Movement Sciences, Department of Pediatrics, Chicago, Illinois, United States; b Federal University of São Carlos, Department of Physical Therapy, São Carlos, Brazil; c National Institutes of Health, Clinical Center, Functional and Applied Biomechanics Section, Rehabilitation Medicine Department, Bethesda, Maryland, United States

**Keywords:** cerebral palsy, hemiplegia, diplegia, brain imaging, functional near-infrared spectroscopy, motor control

## Abstract

**Significance:** Our study is the first comparison of brain activation patterns during motor tasks across unilateral cerebral palsy (UCP), bilateral cerebral palsy (BCP), and typical development (TD) to elucidate neural mechanisms and inform rehabilitation strategies.

**Aim:** Cortical activation patterns were compared for distal upper and lower extremity tasks in UCP, BCP, and TD using functional near-infrared spectroscopy (fNIRS) and related to functional severity.

**Approach:** Individuals with UCP (n=10, 18.8±6.8  years), BCP (n=14, 17.5±9.6  years), and TD (n=16, 17.3±9.1  years) participated in this cross-sectional cohort study. The fNIRS was used to noninvasively monitor the hemodynamic response to task-related cortical activation. The block design involved repetitive nondominant hand squeezing and ankle dorsiflexion.

**Results:** Individuals with UCP demonstrated the highest levels of activation for the squeeze task (UCP>BCP q=0.049; BCP>TD q<0.001; and UCP>TD q=0.001) and more activity in the ipsilateral versus contralateral hemisphere. Individuals with BCP showed the highest levels of cortical activation in the dorsiflexion task (BCP>UCP q<0.001; BCP>TD).

**Conclusions:** Grouping by CP subtype and manual function or mobility level demonstrated significant differences from TD, even for individuals with the mildest forms of CP. Hemispheric activation patterns showed hypothesized but nonsignificant trends.

## 1 Introduction

Cerebral palsy (CP) is the most prevalent child-onset motor disorder and is caused by a nonprogressive brain injury early in life.^1^^,^^2^ Muscle weakness,^3^^–^^5^ impaired selective motor control,^2^^,^^6^^,^^7^ and hyperreflexia^8^^,^^9^ are common impairments in spastic CP, which can lead to secondary changes in muscle, bones, and neural pathways. Reduction in selective motor control is most closely associated with damage to corticospinal pathways^10^^,^^11^ and is a critical contributor to motor disability.

Brain injuries in CP can occur exclusively or mostly on one side (unilateral) or on both sides of the brain (bilateral). Although the central nervous system (CNS) has a remarkable ability to self-organize following an insult early in life, there are complex interactions between the CNS damage, motor experiences early in life, and the child’s physical growth and development that shape individual neurological and clinical outcomes. Neuroimaging and transcranial magnetic stimulation studies in individuals with unilateral cerebral palsy (UCP) have demonstrated that the less damaged hemisphere may retain and utilize developmentally transient ipsilateral cortical projections to the spinal cord and paretic arm.^12^^–^^15^ In the case of bilateral cerebral palsy (BCP), cortical contributions are likely to be altered in a different way to accommodate to injury on both sides. Lateral shifts on the motor homunculus,^16^^,^^17^ which may be due to enlarged ventricles or greater reliance on proximal muscles^18^ and overlapping connections to the upper and lower extremities,^19^ appear to be potential mechanisms of abnormal brain organization in BCP.

Investigations of the cortical mechanisms underlying motor incoordination can be limited in CP when using techniques such as magnetic resonance imaging (MRI), particularly for those with greater neurological or functional severity who may exhibit excessive involuntary movements or exaggerated startle reflexes. We have previously investigated cortical activity in the upper^20^ and lower^21^ extremities using functional near-infrared spectroscopy (fNIRS),^22^ a technique that has several advantages for studying this population. Similar to fMRI, this noninvasive imaging method calculates a hemodynamic response to motor activity and therefore infers areas of the cortex that are active during a particular task. However, in contrast to MRI, it uses light to monitor levels of both oxygenated and deoxygenated hemoglobin (Hb). The fNIRS allows the participant to be in an upright position in a laboratory or clinical setting while performing more functionally relevant tasks and is less susceptible to motion artifacts because the sensors move with the head.

The goal of this study was to evaluate and compare the cortical responses to two distal extremity motor tasks, hand squeezing and ankle dorsiflexion, in individuals with UCP, BCP or typical development (TD). Most functional imaging studies on upper extremity movements have been focused primarily or exclusively on those with UCP, so we were interested in whether the activation patterns for the hand task in BCP would be more similar to those with UCP or TD. Conversely, few if any studies have examined brain activation during lower extremity movements in those with UCP, so we were interested in how similar or different their brain activation patterns during ankle dorsiflexion were to those with BCP or TD. Previously, we have demonstrated that the extent and magnitude of brain activation was greater in upper extremity (UE) tasks in UCP compared to TD^20^ and was more pronounced with greater levels of hand impairment. Similarly, greater extent and magnitude of brain activation was found in lower extremity (LE) tasks in BCP compared to TD^21^ and was more pronounced in poorer gross motor function. What remains unknown and will be addressed in this study is the comparison between those with UCP to BCP on tasks that involve each of the extremities to evaluate similarities and differences in extent, magnitude, and the balance of activation across hemispheres. We hypothesized that brain activation for the more impaired upper and lower limbs in UCP would show a higher degree of ipsilateral brain activation than seen in either BCP or TD. For those with BCP, we hypothesized that brain activation patterns for both unilateral tasks would show a higher degree of bilateral activation than seen in UCP or TD. We further hypothesized that those with poorer motor function in the extremity being evaluated, as assessed by functional classification levels, validated self- or parent-report questionnaires, and a measure of selective control for the lower extremity task, would demonstrate greater differences from TD in brain activation outcomes.

## 2 Methods

### 2.1 Participants

A total of 40 participants were consented into the protocol and completed the study, including 14 (five males) with BCP, Gross Motor Function Classification System (GMFCS) levels I to III, 10 (two males) with UCP, and 16 (nine males) with TD. Participants were included if they were at least 5 years old, able to understand and follow simple directions for performing a repetitive task, and agreed to not drink caffeine or alcohol for 24 h before assessments to avoid associated alterations in blood flow dynamics. Those in the UCP group had unilateral injuries confirmed by structural MRI. Exclusion criteria included any health condition or diagnosis other than CP that would affect the ability to maintain attention or move a body part repetitively for short periods of time, surgery within a year, or botulinum toxin injections within 6 months. All participants, or their parents, as applicable, completed informed consent and assent for those <18  years of age.

Standardized clinical assessments were performed including the GMFCS,^23^ Manual Ability Classification System (MACS),^24^ Hypertonia Assessment Tool,^25^ and Edinburgh Handedness Inventory with an additional question about lower extremity preferences to determine which leg to test. Furthermore, the ABILHAND-Kids,^26^ ABILOCO-Kids,^27^ and Pediatric Evaluation of Disability Index–Computer Adaptive Test (PEDI-CAT) questionnaires (version 2.5)^28^ were completed. Parents or adult participants completed the ABILOCO-Kids and ABILHAND-Kids questionnaires about difficulty performing ambulation and manipulations tasks, respectively. Answers from each question are input to an online algorithm for transformation into logit score ranging from −6 to 6 for linear representation of scores. The PEDI-CAT yields summary scores for mobility and daily activity domains based on adaptive administration of questions. Higher scores indicate better function.

### 2.2 Setup

**Fig. 1:**
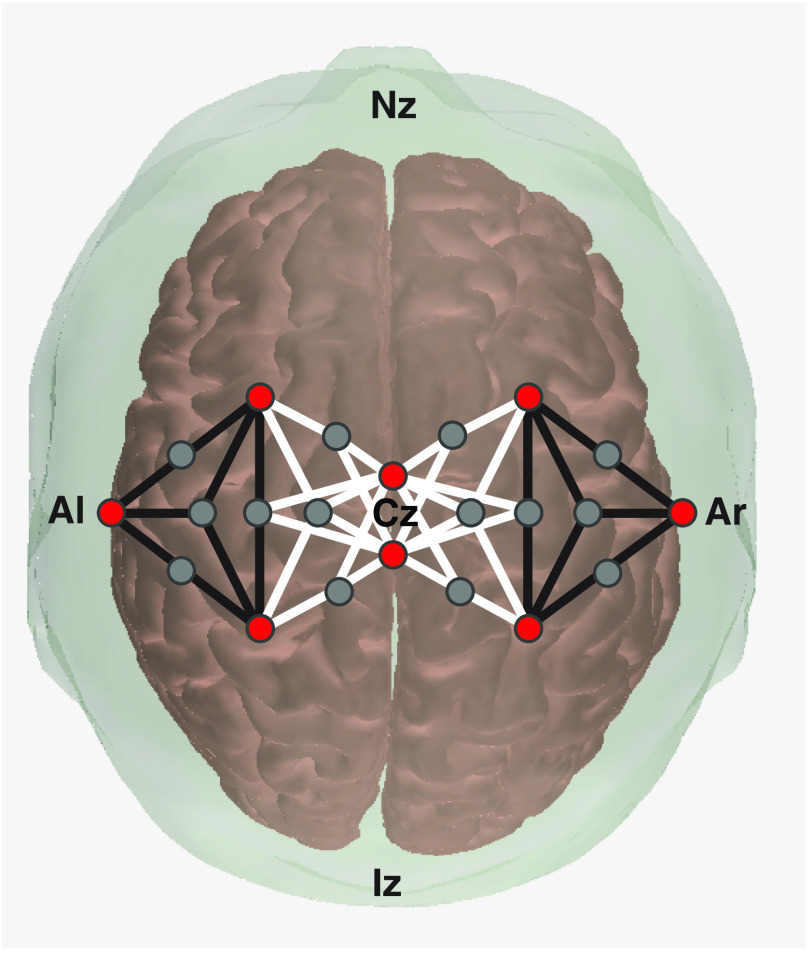
Optode arrangement placed directly on scalp locations over the sensorimotor cortex, as viewed from above. Red circles denote light sources, gray filled in circles denote light detectors, and the lines between them are the channels that represent activation on the cortical surface between the emitter–detector pairs. Channels that were included in the lateral region of interest are denoted in black, while those considered to be medial region of interest are in white. Nz, nasion; Iz, inion; Al, left auricular; Ar, right auricular.

Setup for each participant included placement of the fNIRS optodes (CW6, TechEn, Milford, Massachusetts) on the scalp regions that overlay the sensorimotor cortical areas (Fig. 1). The hair was carefully parted to align with optode placement as described previously.^22^ Optode arrangements differed slightly for UCP and BCP participants, but a common set of channels was identified for all tasks which included eight sources and 14 detectors with a total of 42 channels.

### 2.3 Motor Tasks

The tasks investigated for this cohort were nondominant hand grasp and relaxation around a soft object (“squeeze” task) and nondominant ankle dorsiflexion and relaxation against gravity (“dorsiflexion” task). Participants were seated on a plinth with trunk support and completed eight task blocks of 15 s each, interspersed with a variable rest period of 25 to 35 s. They were cued to move at 1 Hz with audio^29^ and visual prompts customized for this experiment. Participants were video recorded during the tasks to aid in interpretation of data and were given breaks in between tasks as needed.

### 2.4 Data Analysis

**Fig. 2:**
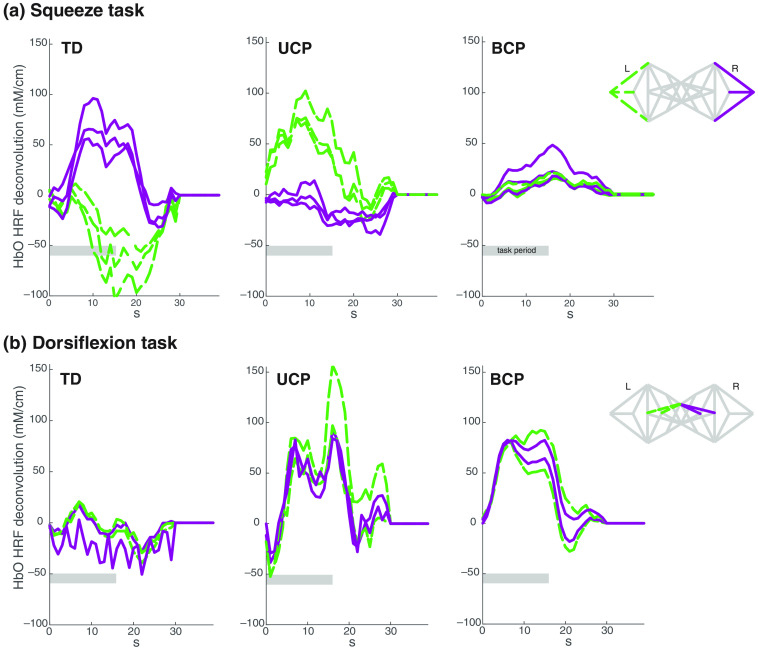
Selected channels for representative participants (n=1 per plot) in each group and task. Lines represent the deconvolution of the impulse response function subject-level analysis in the NIRS toolbox, where purple solid lines are from the right hemisphere, contralateral to the task, and green dashed lines are channels from the left hemisphere, ipsilateral to the task. More medial channels are represented in the dorsiflexion task, and lateral channels for the squeeze task to reflect the relative location on the motor homunculus for these two body areas. The 15-s task period is represented by the gray bar on each graph.

Light intensities in the fNIRS signals were analyzed using a set of commands in the NIRS toolbox.^30^ In brief, the processing steps included converting raw data to optical density, followed by conversion to oxygenated and deoxygenated Hb using the modified Beer–Lambert law. A deconvolution of the impulse response function was visualized using the *<MyStats>.HRF* function in the toolbox,^31^ shown in Fig. 2 as representative signals. Group-level statistical analysis was completed using a first-level canonical general linear model,^30^^,^^32^ which was used to estimate the statistical response of each fNIRS light emitter–detector pair (channel) to the functional task. An autoregressive whitening filter was iteratively computed and applied to the data and linear regressor model to reduce the effect of serially correlated noise.^32^ A robust linear regression using the Huber bisquare weighting was applied to estimate the model. The regression beta coefficients and t-statistical effects were estimated for each fNIRS channel and task condition. This procedure was previously shown to correct the high false-discovery rate problems that result from motion and nontask coupled superficial physiological noise in fNIRS analysis.^32^ For all analyses, the sum of oxygenated and deoxygenated Hb or the total hemoglobin (HbT) was used to consider both aspects of neurovascular coupling and also for its robustness against artifact from blood flow in the superior sagittal sinus which is important when investigating a task such as dorsiflexion.^33^ Statistical estimates were Benjamini–Hochberg corrected for comparisons across all fNIRS emitter–detector pairs and task conditions and the false discovery rate (FDR) corrected p-values (denoted as the q-value) were used.

### 2.5 Evaluation of Hemodynamic Response and Task Performance

To assess differences in brain activity between groups (TD, BCP, and UCP), channels were reversed across the midline for the participants whose tested, functionally nondominant limb was on the right. Therefore, brain hemisphere contralateral to activity was always displayed as being on the right side and was the lesioned hemisphere for those with UCP. To address the primary question of differences between types of CP, the NIRS toolbox was used to test the effect of group on recorded activity, and to evaluate contrasts within the subgroups of CP diagnoses (groups, GMFCS levels, and MACS levels). Secondarily, the contrast between squeeze and dorsiflexion tasks was evaluated within each group. A region of interest analysis was used to distinguish the medial and lateral portions of left and right hemispheres (shown in Fig. 1) to describe topography of activation differences associated with upper and lower extremity tasks.

On an individual participant level, the number of fNIRS channels that was significantly active (q<0.05) and the sum of beta values for all active channels were determined for each hemisphere and across both hemispheres as an indication of the extent and level of brain activity, respectively.

Video recordings were reviewed for the absence or presence of mirror movements observed on the limb opposite to the intended motion. Participants were considered to demonstrate no mirror movements if none were observed, mild mirror movements if analogous movements were observed on the contralateral side <50% of the time, and strong mirror movements if analogous movements were observed on the contralateral side more than 50% of the time.

### 2.6 Laterality Index to Evaluate the Balance of Activation Across Hemispheres

On an individual participant level, laterality indices (LI) were calculated using the difference in beta values of significantly active channels (q<0.05) between hemispheres divided by the sum. This index quantifies the overall balance in activation between contralateral and ipsilateral hemispheres relative to the task being performed and has been widely used in previous studies. A value closer to 1 indicates more strongly contralateral hemisphere activity, while a value closer to −1 indicates more strongly ipsilateral hemisphere activity. Values close to 0 indicate that the two hemispheres were activated to a similar degree during a unilateral task (bilateral activation).

### 2.7 Relationship to Functional Scales

Because development of handedness is likely to be altered by early brain injury, we evaluated if there was a systematic impact of Edinburgh scores with clinical scales. In addition, we performed a range of correlation analyses to determine if relationships existed between fNIRS-derived metrics and clinical examination metrics in participants with CP. Variables derived from the fNIRS analysis (LI, number of active channels on either or both hemispheres and sum of beta values in individual or both hemispheres) were correlated using a Pearson’s correlation with ABILHAND and PEDI-CAT daily activity domain for the squeeze task, and with the ABILOCO and PEDI-CAT mobility domain for the dorsiflexion task. A Spearman correlation was performed relating the Selective Control Assessment of the Lower Extremity (SCALE) and GMFCS to the dorsiflexion task fNIRS metrics and the MACS to the hand squeeze task fNIRS metrics.

### 2.8 Statistical Analyses and Comparisons Between Tasks

The first set of comparisons between tasks was completed using a contrast in the NIRS toolbox between hand and ankle as described above, which allowed for a region-of-interest-based evaluation of cortical activity differences between a hand and ankle task. In addition, a separate one-way analysis of variance was used to evaluate the effect of group (three levels: TD, UCP, and BCP) on each of the following dependent variables: number of active fNIRS channels, sum of beta values for active fNIRS channels, and LI. Significant main effects were explored further using posthoc tests with Bonferroni corrections.

For all statistical tests performed outside of the NIRS toolbox using SPSS (version 26), p<0.05 was considered significant. For any tests done using NIRS toolbox, an FDR corrected p-value, q<0.05, was used as a threshold for significance.

## 3 Results

### 3.1 Participant Characteristics and Clinical Features

Participant demographics are summarized in Table 1. Age did not differ significantly across groups. All participants with BCP had spasticity (HAT item 3) in at least one LE (including the side tested), with five having spasticity in the UE as well. There was spasticity present in both lower and upper extremities of the more impaired side for all with UCP. There were no significant differences in clinical exam scores, with the exception of the BCP group having lower PEDICAT mobility scores. Because light transmission can be decreased by darker hair color, summary participant characteristics^34^ are also reported in Table 1.

**Table 1:** Participant characteristics.

	BCP	UCP	TD	p
Number of participants	14	10	16	—
Age (years)	17.5±9.6	18.8±6.8	17.3±9.1	0.774
Handedness	8 R	3 R	15 R	—
	6 L	7 L	1 L	
GMFCS	I n=4	I n=4	N/A	—
	II n=7	II n=5	—	
	III n=3	III n=1	—	
MACS	I n=8	II n=8	N/A	—
	II n=6	III n=3	—	
ABILOCO (logit)	1.78±1.84	3.17±2.59	N/A	0.079
ABILHAND (logit)	3.48±2.57	3.47±1.44	N/A	0.978
SCALE for more impaired LE	4.4±1.7	4.1±1.5	N/A	0.700
Mirror movements (squeeze)	None n=14	None n=2	None n=16	—
	Mild n=0	Mild n=6	Mild n=0	
	Strong n=0	Strong n=2	Strong n=0	
Mirror movements (dorsiflexion)	None n=5	None n=4	None n=16	—
	Mild n=7	Mild n=2	Mild n=0	
	Strong n=2	Strong n=4	Strong n=0	
PEDICAT mobility	64.4±3.4	67±3.5	N/A	0.044*
PEDICAT daily activity	59.2±6.2	57.9±4.0	N/A	0.685
Hair color	Black n=4	Black n=1	Black n=7	—
	Blonde n=2	Blonde n=1	Blonde n=4	
	Brown n=7	Brown n=9	Brown n=4	
	Red n=1	Red n=0	Red n=1	

BCP, bilateral cerebral palsy; UCP, unilateral cerebral palsy; TD, typical development; GMFCS, Gross Motor Functional Classification System; MACS, Manual Ability Classification System; SCALE, Selective Control Assessment of the Lower Extremity; LE, lower extremity; PEDICAT, Pediatric Evaluation of Disability Index Computer Adaptive Test; N/A, nonapplicable; p, p-value for the statistical comparison between groups; and all values are listed as average±standard deviation.

* Statistically significant.

### 3.2 Evaluation of Hemodynamic Response in both Sensorimotor Cortices

The primary aim of this study was to compare and contrast cortical activation patterns in children with unilateral and BCP, with both compared to those in children with TD, in an upper and lower extremity tasks. Data are further presented across functional classification levels within the CP subtypes, using the MACS for the squeezing task and the GMFCS for the dorsiflexion task.

**Fig. 3:**
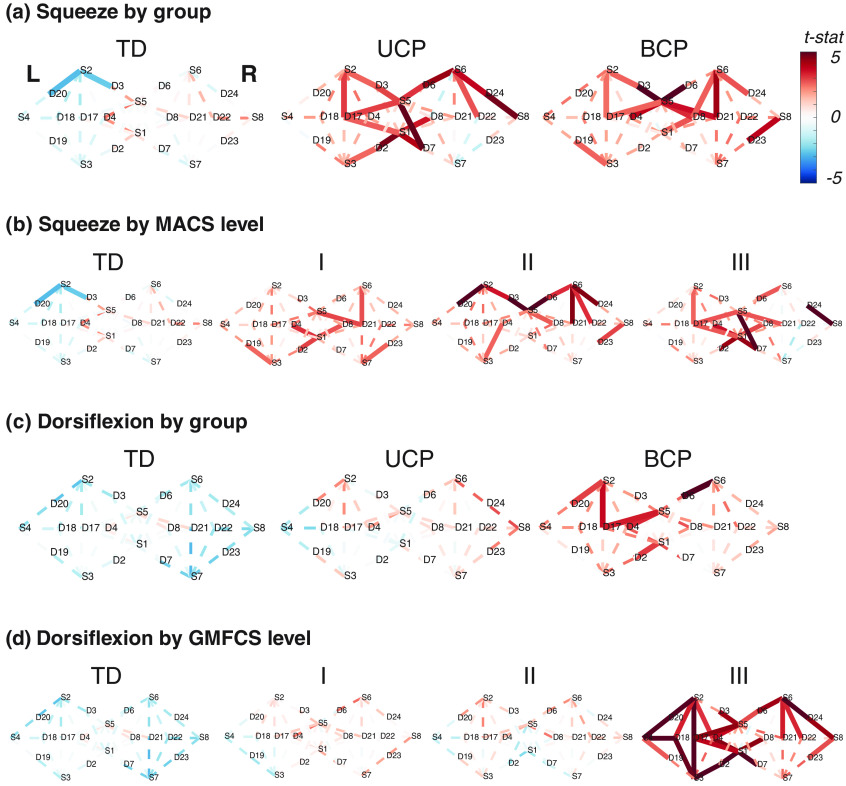
HbT activation maps by group and task. Significantly active channels (q<0.05) are in solid lines, and the colorbar represents the value of the t-statistic (t-stat). Positive values (red colors) denote increases in HbT relative to baseline, and negative values (blue colors) indicate that signal was greater during baseline than task. The right hemisphere is contralateral to the task limb, and the orientation of the probe is centered around Cz with left and right hemispheres denoted by “L” and “R” in the typically developing (TD) map in (a).

For the nondominant hand squeezing task, there was globally more activity in the groups with CP, compared to the group with TD [Fig. 3(a)]. The TD group demonstrated few significantly active channels in group analysis, including two ipsilateral channels that demonstrated a significant reduction in cortical activity relative to rest (negative t-statistic). Although not significant, there were positive t-statistics (indicating cortical activation) on the contralateral hemisphere. Both the UCP and BCP groups had more significantly active channels in both hemispheres than TD, which were all indicative of cortical activity (positive t-statistics). In the contrast analysis, the UCP group had the highest level of activity, with significant differences found between all groups (UCP>BCP q=0.049; BCP>TD q<0.001; and UCP>TD q=0.001). Within the contrasts for TD and MACS levels, the group with the greatest impairments had the highest levels of cortical activity [Fig. 3(b)]. In this analysis, all contrasts were significant except for the comparison between MACS I and II (III>I q<0.001; I>TD q<0.001; III>II q<0.001; II>TD q<0.001; III>TD q<0.001; and I=II q=0.84).

There was also globally more activity in the CP groups for the dorsiflexion task, compared to the TD group [Fig. 3(c)]. Again, there were no significantly active channels in the TD group, but positive t-statistic values were identified relatively close to midline on the contralateral hemisphere. Both CP groups had positive t-statistics in both hemispheres, but the only significantly active channels were found in the BCP group. In the contrast analysis, the highest activity was recorded in the BCP group (BCP>UCP q<0.001; BCP>TD q<0.001; and UCP>TD q=0.001). Within the contrasts for TD and GMFCS levels [Fig. 3(d)], all contrasts were significant (q<0.001) except for the comparison between GMFCS I and II (III>I, I>TD, III>II, II>TD, III>TD, and I=II).

**Fig. 4:**
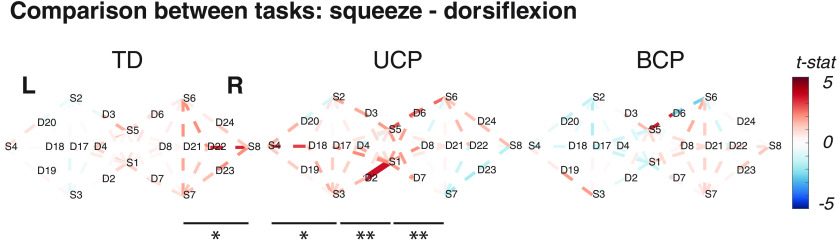
HbT contrast between tasks. Significantly active channels (q<0.05) are shown in solid lines, and the colorbar represents the value of the t-statistic (t-stat). Positive values (red) indicate higher activity level for the squeeze task, while negative values (blue) indicate higher activity level during the dorsiflexion task. Four regions of interest were evaluated (left lateral, left medial, right medial, and right lateral), and significantly different regions are labeled with horizontal bars. *q<0.05, **q<0.001.

When contrasting the upper and lower extremity tasks (Fig. 4), there were almost no significantly different channels in any group. In general, there are more positive than negative t-statistics, which indicate greater activity during the upper extremity squeeze task than the lower extremity dorsiflexion task in all groups. In the TD group, this difference was found to be significant when the channels on the lateral portion of the contralateral hemisphere (defined in Fig. 1) were grouped in a region of interest (q<0.001).

In the UCP group, there were higher levels of activity during squeeze compared to dorsiflexion across of the ipsilateral hemisphere (lateral ipsilateral q=0.01 and medial ipsilateral q<0.001), and in the medial aspect of the contralateral hemisphere (q<0.001). In contrast, the lateral portion of the contralateral hemisphere was not differently active between tasks (q=0.89).

There were no statistically significant differences between squeezing tasks and dorsiflexion tasks in the BCP group when considering any channel individually or portions of either hemisphere as a group (all regions q=0.91).

### 3.3 Evaluation of Group Differences in Which Hemisphere Had Greater Brain Activation

**Fig. 5:**
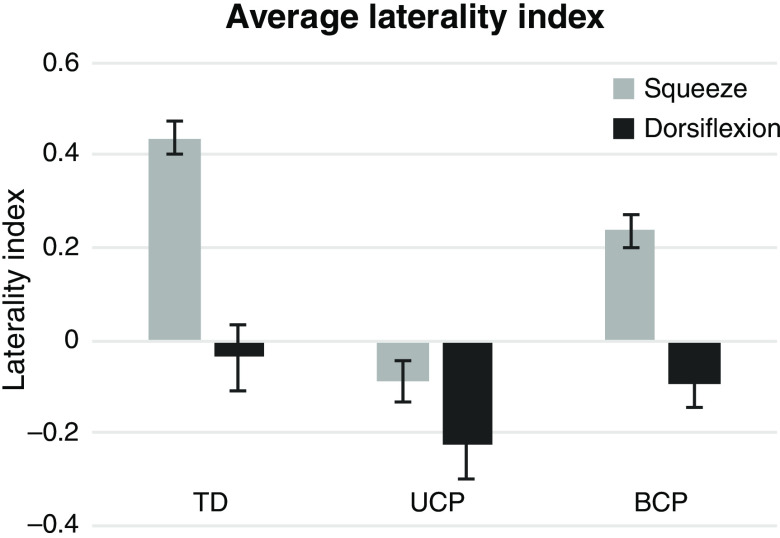
Average of the laterality index (LI) across participants, with standard error bars. A positive LI value indicates more activity on the contralateral hemisphere to the task, while a negative LI indicates higher ipsilateral activity with values centered around 0 indicating more bilateral activation patterns. For squeezing, there was a trend of an effect of group (F=2.842, p=0.076). For dorsiflexion, there was no effect of group (F=0.812, p=0.452). TD, typically developing; UCP, unilateral cerebral palsy, and BCP, bilateral cerebral palsy.

We anticipated that the group with UCP would demonstrate more ipsilateral brain activity especially when using their nondominant upper limb than those with BCP or with TD. For the LI, there was a nonsignificant trend toward an effect of group (F=2.842 and p=0.076) in the squeezing task (Fig. 5), where both TD and BCP demonstrated a contralateral response on average, while the UCP group brain activation was shifted toward the ipsilateral hemisphere. There was no effect of group (F=0.812 and p=0.452) in the dorsiflexion task where all groups tended toward a bilateral or slightly ipsilateral response, with the UCP group being the most ipsilateral on average, and the BCP group value between the UCP and TD values.

Another way to assess hemispheric differences was to compare brain activation outcomes in the hemisphere ipsilateral versus contralateral to the limb performing the task. There were differences found in the number of active channels and beta values from the GLM in that the UCP group had the highest values in the squeeze task, while the BCP group was highest in the dorsiflexion task as stated previously. The sum of significant beta values was used as a measure of intensity of activity, and there was a main effect of group for both the ipsilateral (F=4.423; p=0.023; UCP>TD p=0.051; and UCP>BCP p=0.053) and contralateral (F=5.031; p=0.014; UCP>TD p=0.018; and UCP>BCP p=0.051) hemispheres during the squeeze task. The extent of activity is evaluated by the total number of active channels in each hemisphere. Both CP groups were higher on average than TD. There was a main effect of group for the ipsilateral hemisphere during the dorsiflexion task (F=4.051, p=0.026; BCP>TD, p=0.022).

### 3.4 Relationship to Functional Scales

No correlation was found between fNIRS variables (beta LI, active channels on either or both hemispheres, beta in either or both hemispheres) during the squeeze task with ABILHAND, PEDI-CAT daily activities, or mirror movements. The beta in the right hemisphere during dorsiflexion was inversely related with the ABILOCO (rho=−0.467, p=0.044). There were no other correlations found between fNIRS variables (beta LI, active channels on either or both hemispheres, beta in either or both hemispheres) during the dorsiflexion task with ABILOCO, PEDI-CAT mobility score, or mirror movements.

There was, however, a significant relationship between the MACS level and number of active left hemisphere channels (rho=0.366, p=0.02), total number of active channels (rho=0.352, p=0.03), and beta in the left (rho=0.635, p<0.001), right (rho=0.459, p<0.01), and both (rho=0.567, p<0.001) hemispheres during the squeeze task.

There was also a significant relationship between the GMFCS level and number of active left (rho=0.437, p<0.01), right (rho=0.432, p<0.01), and both (rho=0.467, p<0.01) hemisphere channels, and beta in the left (rho=0.559, p<0.001), right (rho=0.594, p<0.01), and both (rho=0.643, p<0.001) hemispheres during the dorsiflexion task. There was a trend noted for a relationship between SCALE score and beta across both hemispheres (rho=−0.397, p=0.058) and beta on the left hemisphere (rho=−0.452, p=0.060), indicating that higher activity may be associated with poorer selective motor control in CP.

## 4 Discussion

We evaluated the activity of the sensorimotor cortical areas during focal, distal tasks of the upper and lower extremities in individuals with bilateral and unilateral CP. All of whom were GMFCS and MACS levels Ito III and able to perform the fairly simple tasks included here with their more impaired upper and lower extremity. Both groups with CP were also compared to a same age cohort without CP (TD). There were notable differences in the extent of brain activity, as measured here by number of significantly active channels, between the groups with CP and those that did not have CP. Results for CP subtypes varied by task. As hypothesized, the greatest extent of activity across the brain for the hand task was in the group with UCP and for the ankle task was in the group with BCP. In this cohort, the MACS level-I group only included those with BCP, and the MACS level-III group included only those with UCP, indicating poorer hand function in UCP as a group. This result is also consistent with the observed research emphasis on the UE in UCP^35^^,^^36^ and in LE tasks such as gait in BCP.^37^^,^^38^ Movement characteristics such as mirroring, which was detected in the participants with both UCP and BCP, may impact the measured cortical activity with both sides being active at the same time. Techniques have been proposed to manage this,^39^ including by our own group,^20^^–^^22^ but they require additional monitoring of the motion of the extremities on both sides of the body not consistently available in this data set.

In contrast to the UE, the UCP group overall demonstrated high skill levels in gait seen with an average higher ABILOCO and PEDI-CAT mobility scores compared to BCP. Despite this greater community mobility, they still had significantly higher cortical activity associated with LE efforts compared to those with TD, consistent with reports from walking tasks on a treadmill.^40^ When CP subtypes were redistributed into their respective GMFCS and MACS levels for comparison of cortical activity based on the severity of functional impairment, each classification system level was significantly different from TD and level III was worse than the other two levels in both cases (Fig. 2). Results of the contrast between squeezing and dorsiflexion (Fig. 3) showed that the group with UCP had a greater number of active channels in the UE task most prominently on the ipsilateral side, with no differences noted in BCP. This indicates greater modulation of the ipsilateral hemisphere, especially for tasks that were more difficult as evidenced by greater MACS impairment in the UCP group. These tasks are simple for individuals without brain injuries, as seen by the very few significantly active channels in the TD group, with some evidence of possible inhibition as shown by some significant deoxygenated hemoglobin levels on the opposite (ipsilateral) hemisphere in the squeeze task. Brain activation patterns for all children with CP, even in the highest functional levels, differs significantly from those with TD.

For many children with CP, selective voluntary motor control is most impaired distally.^18^ Poorer selective control may be related to greater spread of cortical activity in cases where other more proximal muscles within the same limb, or homologous muscles on the contralateral limb, are coactivated when attempting to perform a focal single joint movement.^21^ Both CP groups had similar SCALE scores and a high percentage of those with mirror movements in the lower extremities; however, only those in the group with UCP had upper extremity mirroring. These clinical evaluations demonstrate that multiple muscles were activated in addition to the ones needed for the task. This is consistent with our earlier studies showing that muscle effort is related to brain effort or activity,^20^^,^^22^ and early indications from gait in BCP.^41^

The contribution of this paper is the comparison across two subtypes with CP and to those with TD of which hemisphere is more active during each of these tasks when the most impaired limb is performing it. We hypothesized that there would be a greater tendency for a more ipsilateral activation pattern in UCP and for a more bilateral activation pattern in BCP compared to the other subtype and to TD. No significant differences were found; however, there was a trend toward more bilateral or ipsilateral activation in UCP compared to a contralateral pattern in both TD and BCP, although BCP was less strongly contralateral on average. Dorsiflexion interestingly was not prominently contralateral even in TD, which is likely related to the fact that the location of ankle representation on the motor cortex is very close to the midline of the head, and the spatial precision of fNIRS not as precise as fMRI. Therefore, the small mean differences shown here, while interesting, require further investigation with imaging techniques with better spatial resolution,^42^^,^^43^ or perhaps multimodal approaches,^44^ for greater confidence in the ability to detect true differences across hemispheres in this or similar tasks.

fNIRS is a promising mobile brain imaging technology for assessing task-related cortical activation patterns in CP and other brain disorders at a group level. The low signal-to-noise ratio and the differences across individuals with CP in how their brain self-organizes in response to a developmental brain injury^45^ make it difficult to use fNIRS on an individual level for intervention purposes, but the field is progressing rapidly toward this goal. Other limitations in this study were the small sample size that made it difficult to find hemispheric differences in activation patterns. Also, we included all children diagnosed with UCP based on their functional profile and structural MRI rather than on etiology. Those with neonatal stroke may show a more uniform pattern of activation. However, the location and extent of the stroke also could have a major effect on brain organization and outcomes, as has been shown in adult stroke.^46^^,^^47^ Some differences were still evident across CP subtypes here, although they may have been more pronounced if inclusion criteria on etiology had been more restrictive.

In conclusion, the comparison of brain activation associated with the same activities in unilateral, bilateral, and no brain injury showed differences that were related to both the distribution of brain injury and the task being completed. Hypotheses about brain laterality were partially confirmed, where UCP demonstrated ipsilateral activation on average for both lower and especially upper extremity tasks. However, this finding was not robustly different when compared to TD and BCP across tasks.

The high neural load for simple single joint tasks highlights the complex mechanisms of movement and activity limitations in CP. This remains an important area of inquiry to motivate interventions and advance our understanding of brain injury in early development.
